# Super-Resolution Real Imaging in Microsphere-Assisted Microscopy

**DOI:** 10.1371/journal.pone.0165194

**Published:** 2016-10-21

**Authors:** Hok Sum Sam Lai, Feifei Wang, Yi Li, Boliang Jia, Lianqing Liu, Wen Jung Li

**Affiliations:** 1 Mechanical and Biomedical Engineering, City University of Hong Kong, Tat Chee Avenue, Hong Kong; 2 State Key Laboratory of Robotics, Shenyang Institute of Automation, Chinese Academy of Sciences, Shenyang, 110016, China; 3 University of Chinese Academy of Sciences, Beijing, 100049, China; Pennsylvania State Hershey College of Medicine, UNITED STATES

## Abstract

Microsphere-assisted microscopy has received a lot of attention recently due to its simplicity and its capability to surpass the diffraction limit. However, to date, sub-diffraction-limit features have only been observed in virtual images formed through the microspheres. We show that it is possible to form real, super-resolution images using high-refractive index microspheres. Also, we report on how changes to a microsphere’s refractive index and size affect image formation and planes. The relationship between the focus position and the additional magnification factor is also investigated using experimental and theoretical methods. We demonstrate that such a real imaging mode, combined with the use of larger microspheres, can enlarge sub-diffraction-limit features up to 10 times that of wide-field microscopy’s magnification with a field-of-view diameter of up to 9 μm.

## Introduction

It has been presumed in the past that even the best optical microscopes in the world would be subjected to a limit in resolution due to the diffractive nature of light. Based on Abbe’s theory, this limit is half of the illumination wavelength [1] (i.e., approximately 200 nm for visible wavelengths). However, during the past two decades, strong efforts have made optically viewing nano-scale features smaller than 200 nm possible. Nobel-Prize-winning fluorescence-based techniques like stimulated emission depletion microscopy (STED) [2] and photo-activated localized microscopy (PALM) [3] are good examples of such methods that can attain live-cell imaging with resolution beyond the diffraction limit. Also, spatially modulated illumination (SMI) can increase the resolution of fluorescence microscopes by constructing a standing wave and reducing the 3D point-spread function of illumination [4]. However, because the intrusive nature of fluorescence microscopy is generally deleterious, developing super-resolution techniques that do not use fluorescence is important. A current trend towards super-resolution images involves developing metamaterials or designing unique lenses that can break the diffraction barrier [5]. However, the current development of such a lens is either limited by fabrication complexity or is limited by high losses within the lens [6]. Conversely, digital reconstruction techniques such as deconvolution [7] are often used as a complement to further increase the image resolution and contrast but also limit the possibility for real-time imaging.

Another significant group of non-fluorescent, super-resolution microscopy techniques is based on collecting evanescent waves near the sample surface. Near-field optical scanning microscopy (NSOM) has experienced rapid development over the past few decades, developing different operating modes such as the transmission, reflection, collection and illumination-collection modes. Sharp probe-tips have also been developed to be either aperture-less or to contain an aperture. Although NSOM can often achieve a resolution of a few nanometers, which is comparable to certain profiling machines, its speed and tip requirement hinders the application of such technology. Then, in 2011, Wang et al. [8] discovered that when placed at close proximity to samples, microspheres could surpass the resolution attained by a solid-immersion lens (SIL). In that study, microspheres were placed on the sample, and a virtual image was observed through the silica microsphere, resolving features down to 50 nm.

The simple and effective approach of microsphere-assisted microscopy has thus attracted strong interest from the research community. The comparison between a solid-immersion lens and microspheres was further strengthened and repeated by Darafsheh et al. [9]. Efforts have also been made to study the mechanism and to further improve its resolution. For example, a group demonstrated that microspheres immersed in isopropyl alcohol have a better contrast plus self-healing properties [10]. There have also been combinations of techniques with microspheres, such as a confocal combination [11] and fluorescence observations [12], that showed improvements in resolution. A functional scanning platform based on microspheres has been created by attaching a microsphere to a glass micro-pipette with a high-precision stage [13].

**Fig 1:**
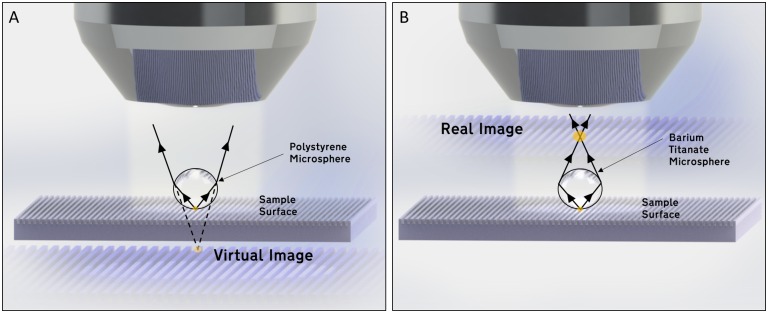
Illustration of super-resolution imaging modes. (A) virtual imaging mode; and (B) real imaging mode.

However, in all studies and applications, the image observed or captured through the microscope was always virtual, as shown in Fig 1(A). The only study that reported on a real image discovered only a blurred real image through a microsphere that was semi-immersed in SU-8 [14]. Also, few theoretical discussions are published in the literature that conclude anything regarding real super-resolution imaging using microspheres. In this study, we report the discovery of such a real-imaging mode, as depicted in Fig 1(B), and share discussions regarding it.

## Methods and Materials

Blank Blu-ray disc (TDK BD-R 25 GB) samples were cut into small pieces with the protection layer of each piece removed. Additionally, a commercially available CPU (central processing unit) chip with 22nm lithography (Intel G2010) was purchased and prepared so that their sub-micron features could be investigated. The heat spreader caps were removed at an elevated temperature of 350°C, and their features were exposed via forced tearing of the silicon chip.

Dry barium titanate microspheres with diameters between 20 and 220 μm, and wet polystyrene microspheres with diameters of 30 μm were acquired from Cospheric and Sigma Aldrich, respectively. The refractive index of barium titanate microsphere and polystyrene microsphere are 1.9 and 1.59, respectively. Deionized water was used to wet the dry microspheres or to dilute the microsphere solution, facilitating dispersion and injection onto the sample’s surface. All samples were placed on a hot plate at 70°C to accelerate evaporation. The microspheres were attached to the sample surface via the electrostatic and van der Waals forces. Each sample was allowed to cool down to room temperature for 10 minutes.

All results were obtained using the Nikon Ti-E, which is an inverted microscope with a motorized stage and a high-precision nosepiece (XY: 100 nm, Z: 25 nm), except for the results of the CPU chip. The results of the CPU chip were obtained using the Nikon Ni-E, which is an upright microscope with the same type of motorized stage and nosepiece, since the CPU chip is too small to be held upside down for observation. All samples were observed in air in reflection mode under bright-field illumination, with the aperture and field diaphragm adjusted mechanically to their minimum size. The diaphragms were minimized to reduce the light scattering from the objective and the microsphere, therefore reducing the washout effect on magnified features by background light and maximizing the contrast. A mercury, pre-centered, fiber illuminator was used with a 425-nm long-pass filter to provide white-light illumination. Vertical scanning was controlled and assisted by Nikon’s Nis-AR software. Andor Zyla 5.5 and Oria 2.0 cameras used to capture grayscale and color images, respectively. The size of the field-of-view of the images captured by a conventional 50x objective for the grayscale and color camera are 266μm×322μm (width × height) and 221μm×221μm, respectively. The field-of-view is the same for both the inverted and upright microscopes. No operations other than cropping and intensity range adjustment were applied to the images reported in this study.

The atomic force microscopy (AFM) image was captured using Autoprobe CP (Park Scientific Instruments) in contact mode with a silicon nitride cantilever with standard tip (Park Scientific Instruments). On the other hand, the scanning electron microscopy (SEM) images were obtained in high vacuum mode using Quanta 250 Environmental SEM (FEI) without coating at 30kV.

## Results and Discussion

**Fig 2:**
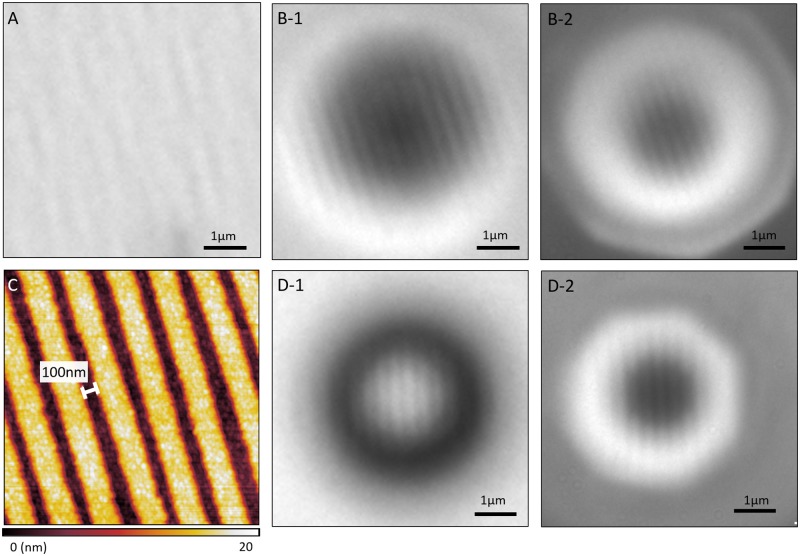
Blu-ray disc sample observed using different methods and conditions. (A) image captured with a 100x (N.A. 0.9) objective lens; (B) image captured with a 30-μm PS microsphere with (B-1) a 20x (N.A. 0.75) objective lens, and (B-2) a 50x (N.A. 0.45) objective lens; (C) results using AFM; (D) image captured with a 25-μm BTG microsphere with (D-1) a 20x (N.A. 0.75) objective lens, and (D-2) a 50x (N.A. 0.45) objective lens. The values on top of each scale bar represents the scale bar’s length that is estimated in real object scale.

According to the literature [8,15–17], a Blu-ray disc has a gap between its tracks that ranges from 100 to 120 nm [8,16,17]. This separation is below the diffraction limit, making it unobservable using normal microscopes and suitable for the super-resolution tests performed in this study. Therefore, we observed a Blu-ray disc’s surface using different methods; the results of these tests are shown in Fig 2. The Blu-ray disc was scanned using AFM and observed through a 100x (N.A. 0.9) objective lens without microspheres. Fig 2(A) shows that a 100x (N.A. 0.9) objective lens cannot resolve the features in the Blu-ray disc without a microsphere. The results obtained using AFM scanning, which are shown in Fig 2(C), verify that the separation between the grooves was close to 100 nm. Consequently, a comparison between Fig 2(B) and 2(D) shows that when in air, a polystyrene bead resolves the Blu-ray disc through a virtual image, while a barium titanate bead resolves it through a real image. Both demonstrate the super-resolution capability of microsphere-assisted microscopy. Real image, as predicted by traditional optics, must be located at the side of the lens (in our case it is a microsphere) opposite to the object/sample while virtual image must be located at the same side of the lens as the object. With the motorized nosepiece of our microscopes, we could therefore determine whether the image captured is located at a position beyond or before the microsphere. In Fig 2(B-1) & 2(B-2), the respective focus positions relative to the sample’s surface are -101μm and -73μm while for Fig 2(D-1) & 2(D-2), the respective focus positions are 72μm and 112.7μm. Negative values indicate that the focus position into the sample’s surface thus confirming virtual image while positive values indicate positions away. Since the BTG microsphere’s size is only 25μm, the image plane of Fig 2(D-1) & 2(D-2) must be located between the microsphere and the objective. Therefore, Fig 2(D-1) & 2(D-2) are real images. Comparing Fig 2(B-2) and 2(D-2), which were both captured using a 50x (N.A. 0.45) objective lens, reveals a similar contrast between both imaging modes. However, the different focusing characteristics of the microspheres with different refractive indices can result in different light uniformities, as shown in the differences between Fig 2(B-1) and 2(D-1).

In addition to this difference, real imaging mode has some distinct advantages over virtual imaging mode. Since the focus position is away from the sample rather than into the sample for real imaging mode, there will be an additional increase to the working distance of the objective. Take for example the barium titanate microsphere with the size of 25μm diameter, using it would allow an additional of 180μm more clearance between the microsphere and objective than using similar size PS microsphere that can only form virtual images. The difference in the focus position, or the additional clearance would be even greater for larger microspheres. A discussion of the focus position of different sizes of microspheres will be presented in later parts of this article. Nonetheless, the additional clearance could be very critical for applications that would need the addition of mechanical arms that can be used to displace the microsphere to specific sites of interest. Such systems have been investigated recently to strengthen the practicality of microsphere-assisted microscopy [13,18]. With larger clearance, high quality or high magnification objectives, which usually have low working distance values can be used even with mechanical arms. Moreover, with real imaging mode, it is now possible to apply a filter (such as adding a pinhole) directly on the real image before the objective. Thus, this mode adds more possibilities for the enhancement of resolution or contrast than virtual imaging mode.

**Fig 3:**
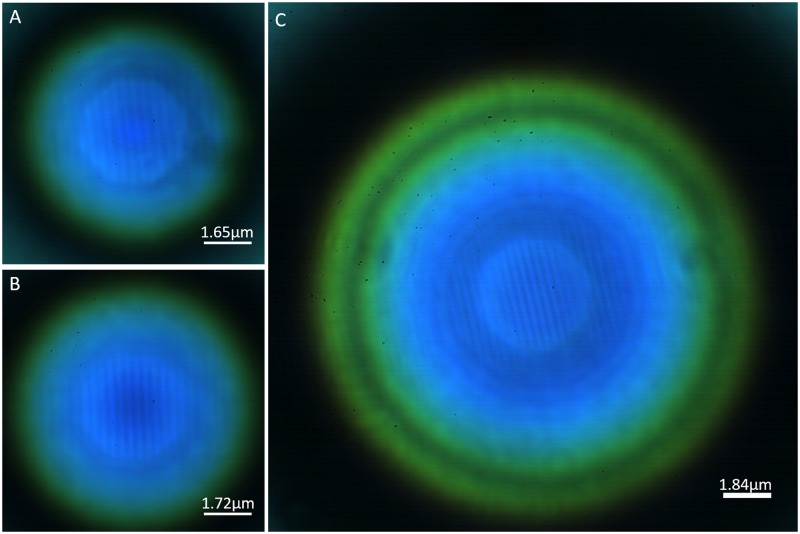
Color images of a Blu-ray disc captured under different conditions. (A) 65-μm BTG, (B) 90-μm BTG, and (C) 175-μm BTG. All images were captured using a 50x (NA 0.45) objective lens. All scale bars represent 20 μm without including the microsphere’s additional magnification. The value on top of each scale bar represents the scale bar’s length that is estimated in the real object scale.

A variety of sizes of barium titanate microspheres was used to observe the Blu-ray disc in air. Fig 3 includes these images, which were captured using a color camera and verify the super-resolution produced with a wide range of sizes of barium titanate microspheres. Although color dispersion occurs at the ring, dispersion is not observed in the center, allowing for useful color information of the object to be collected with super-resolution spatial information. This is an advantage over other super-resolution techniques that require further processing, separate color channels, or pseudo-coloring to obtain a color image.

**Fig 4:**
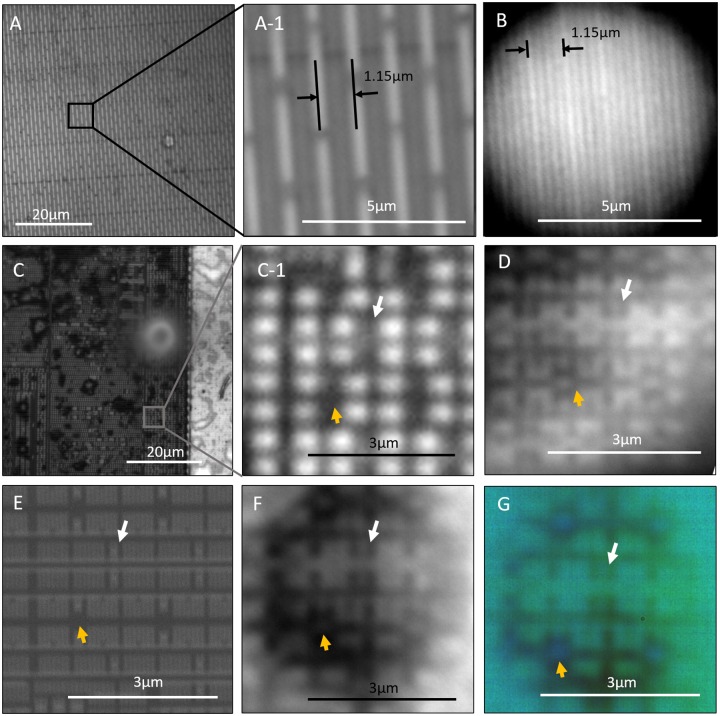
Comparison of images of CPU (22-nm lithography CPU: Intel G2010) features captured in different ways. (A) CPU observed with a 100x (NA 0.9) objective lens, where subset (A-1) shows the magnified area of interest; and (B) the same location of the CPU as in (A) observed through a 200-μm diameter BTG microsphere with a 100x (NA 0.9) objective lens. The smallest gap width resolved in (B) is estimated to be 120 nm wide. (C) a second location observed with a100x (NA 0.9) objective lens, where subset (C-1) shows the magnified area of interest; (D) a 25-μm diameter PS microsphere with a 100x (NA 0.9) objective lens observing on the features in (C); (E) SEM image of the same features in (C) at 30 kV; (F) a 15-μm diameter BTG microsphere with a 100x (NA 0.9) objective lens; and (G) same condition as (F) but captured with a color camera. The value on top of each scale bar represents the scale bar’s length in real object scale. The additional magnification factors for (B), (D), (F) and (G) are estimated to be 6.44x, 6.92x, 5.62x and 6.33x, respectively, using the proposed method mentioned later in this article.

In addition to observing Blu-ray disc samples, we used microspheres with high refractive indices to observe feature-exposed CPU. Comparisons of the images captured with and without a microsphere for repetitive patterns are shown in Fig 4. In the images captured with a microsphere, sharper corners and smaller features were resolved, further indicating the achievement of super-resolution. Consequently, the smallest feature in Fig 4(B) is estimated to be 120 nm wide, which is the gap between the lines; this yields further evidence for the super-resolution of real images that are formed through high-refractive index microspheres.

Using the CPU as sample, a comparison among different imaging modes for microspheres using a conventional microscope and a scanning electron microscope is shown in Fig 4(C)–4(G). In Fig 4, the white arrows refer to two lines that are less than 40 nm wide with a pitch of less than 80 nm. Because these lines are smaller than 40 nm, they are not visible using a conventional microscope, even with an objective lens with a numerical aperture as high as 0.9. However, assisted by a microsphere, the two lines are clearly visible despite the unresolved line gap in both the virtual imaging and real imaging shown in Fig 4(D) and 4(F), respectively. There are no distinct differences in resolution between the virtual and real imaging modes shown in Fig 4(D) and 4(F). Although the image captured through a microsphere has a lower resolution than that of SEM, there are features that SEM cannot distinguish, such as the dot-like feature indicated by the yellow arrows in Fig 4(D), 4(F) and 4(G). Many features within insulating materials or below a transparent dielectric film are exclusive to optical methods; thus, super-resolution optical methods provide useful results.

The super-resolution mechanism of a microsphere was first explained using the concept of optical reciprocity of the photonic nano-jet effect [8]; others explained that super-resolution is due to the microsphere’s ability to collect high spatial frequency signals at near-field distances [19]. Additional factors for increased resolution have also been suggested, such as the whispering-gallery mode effect [20] and localized surface plasmon resonances in nanostructured metallic objects [21]. These theories neither restrict the image to be virtual nor depend on whether the image is virtual. With the similarity between the images obtained using the virtual and real imaging mode, these previous super-resolution mechanisms can also be applied to real images.

However, to further investigate the similarity and differences between the real and virtual imaging mode, we simulated microspheres of two refractive indices and two sizes under the same condition of having a dipole excitation located directly below the microsphere and at a horizontal offset of 500 nm. All dipoles are oriented parallel to paper and are aligned horizontally.

**Fig 5:**
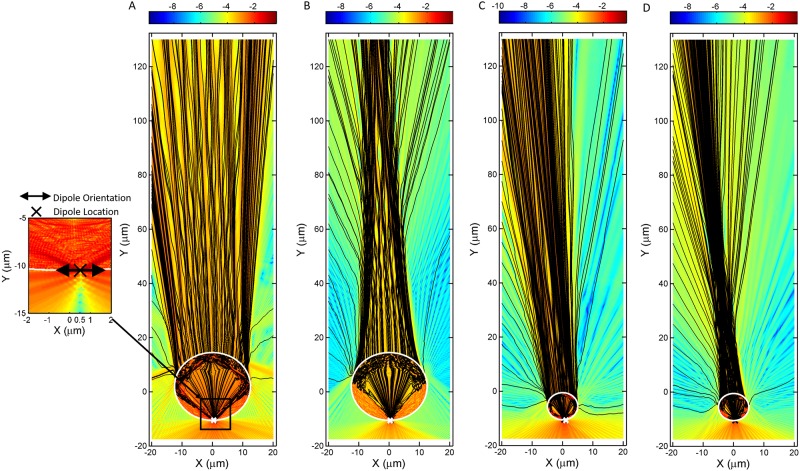
2D simulation using FDTD, displaying Poynting vector magnitudes and directions as colors and streamlines, respectively, for different combination of refractive index n and the size of the microsphere in radius R. (A) n = 1.59, R = 12.5 (B) n = 1.9, R = 12.5 (C) n = 1.59, R = 5 (D) n = 1.9, R = 5. For all conditions, a dipole excitation is located at the coordinate of (0.5, -10), which is of 500nm horizontal offset to the bottommost point of the microsphere at (0, -10). The unit of the coordinates is in micrometer. The location of the dipole excitation and orientation is indicated in the inset in (A).

**Fig 6:**
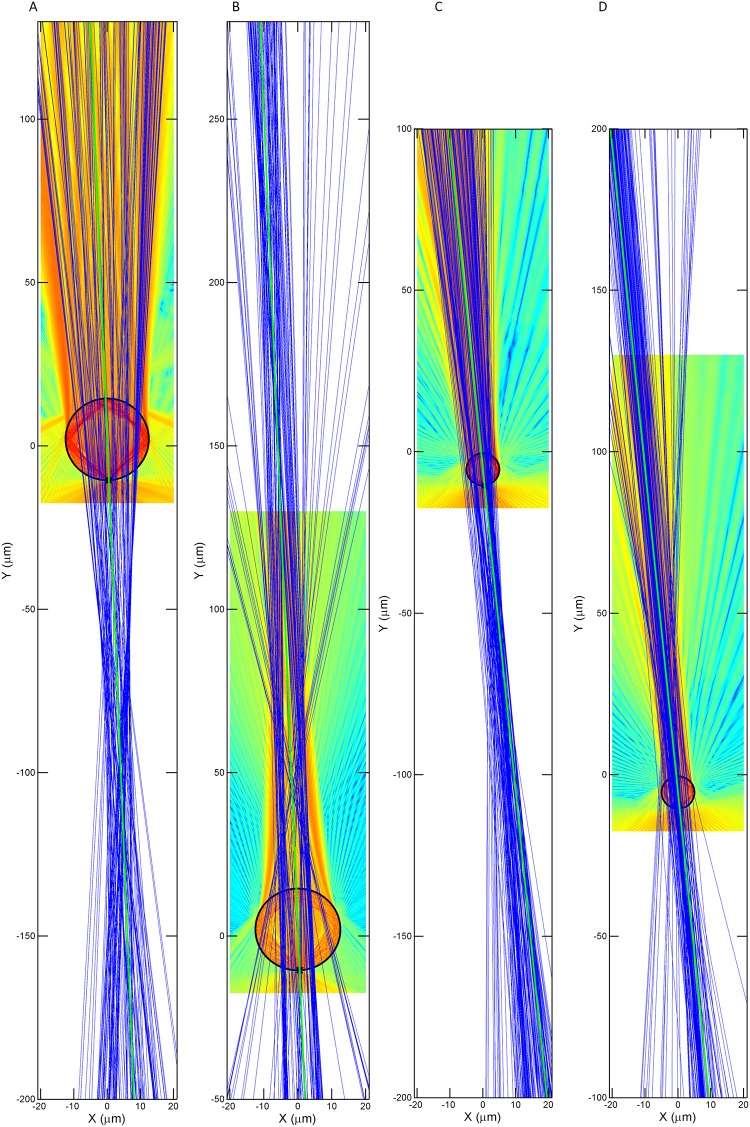
Extrapolated linear fits of the top segments (i.e., segments from 80 to 130 μm) of the Poynting streamlines with different refractive indices and microsphere sizes. (A) n = 1.59, R = 12.5 (B) n = 1.9, R = 12.5 (C) n = 1.59, R = 5 and (D) n = 1.9, R = 5.

We used the FDTD method (Lumerical FDTD Solutions) and ran the simulation in 2D TE mode. By tracing and connecting the Poynting vectors, we obtained the figures shown in Fig 5. The divergence of the Poynting vectors at the top of the simulation domain are shown to increase when the refractive index changes from 1.59 to 1.9. From the simulations with n = 1.59, there are no marked convergence of the streamlines on top of the sphere; when n = 1.9, the convergent areas appearing on top of the sphere correspond to an image spot. On the other hand, a comparison between larger microspheres and smaller microspheres can also be made from Fig 5. Poynting vectors of smaller microspheres lean more to the left as a whole compare to larger microspheres for both refractive indices. Further analyses, which fit the far-field segments (i.e., segments from 80 to 130 μm) of the streamlines into straight lines, are shown in Fig 6. These plots are similar to ray-tracing methods, where converging areas or intersections lying on the axis joining the object with the microsphere’s center can be perceived as image locations. In all of the plots shown in Fig 6, no exact converging points are shown; however, a range of intersection planes are present. The narrower the area of convergence, the higher the image resolution. Comparing the area of convergence in Fig 6(A) with 6(C) and 6(B) with 6(D), we know that a smaller microsphere would be able to attain higher resolution. How these converging areas shift based on the refractive index reconciles with the optical geometries, where the image formation is dependent on the relationship between the object position and the focal length of the lens. As the refractive index of the microsphere increases, the focal spot of the microsphere shifts to be within the microsphere, allowing magnified real images to form if objects are located very near to the sphere. As shown in Fig 6, the converging areas switch sides from virtually beyond the microsphere to above the microsphere as the refractive index increases from 1.59 to 1.9.

**Fig 7:**
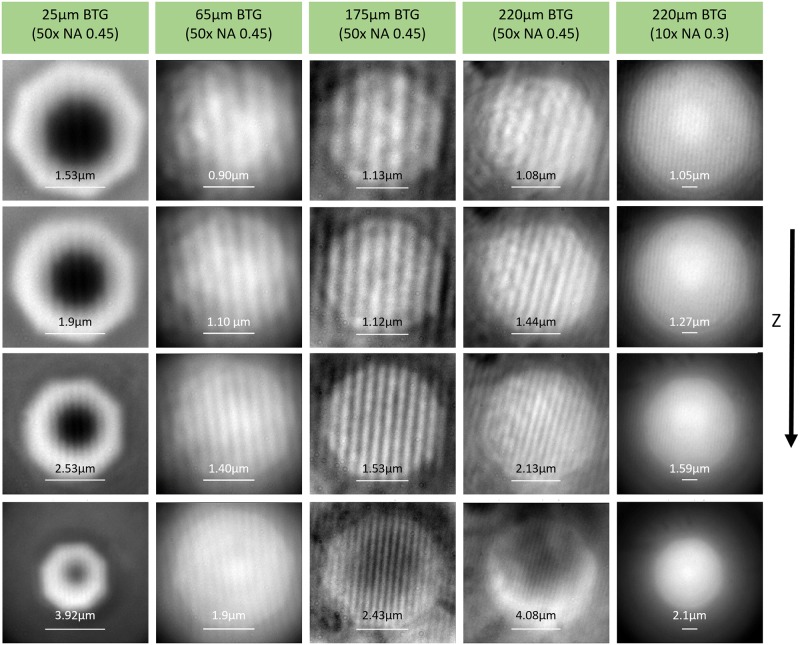
Blu-ray disc samples observed with different microsphere sizes at different focus positions (Z). Focus positions descend with the row they are located in. All scale bars represent 20 μm without including the microsphere’s additional magnification. All images are captured using a 50x (NA 0.45) objective lens, except for the rightmost column. The value on top of each scale bar represents the scale bar’s length that is estimated in the real object scale.

A range of intersection planes in Fig 6 also indicates that there would be a range of image planes for microspheres. This matches the result that more than one focus position can acquire clear images. In fact, when an objective lens’s focus moves to different positions, the images captured are at different magnifications. A vertical scan with the objective lens provides a volume of data that can help confirm the relationship between the effective magnification and focus position. Images taken at different planes using different sizes of microspheres are shown in Fig 7. As the objective lens moves up and away from the microsphere, the real image is magnified more strongly, which is the opposite of the case for a virtual image [22]. This is in accordance with the prediction by simulation in Fig 6. As the objective lens moves past the location that produces the best image sharpness, the image begins to blur, and the contrast and resolution decrease rapidly.

**Fig 8:**
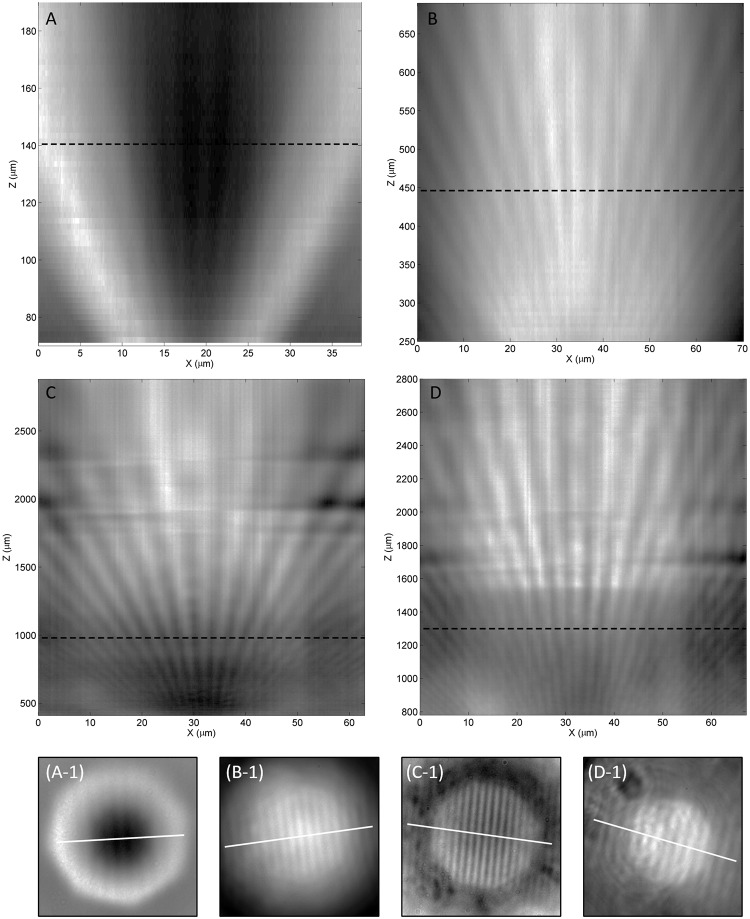
Cross-section slice of z-stacked images captured under different conditions. (A) a 50x (NA 0.45) objective lens with a 25-μm BTG; (B) a 50x (NA 0.45) objective lens with a 65-μm BTG; (C) a 50x (NA 0.45) objective lens with a 175-μm BTG; and (D) a 50x (NA 0.45) objective lens with a 220-μm BTG. Subsets (A-1), (B-1), (C-1), (D-1) correspond to subfigures (A)-(D), where each shows the cutting line that results in the cross-section slice. Similarly, the dotted line in each cross-section slice shows the focus position when capturing the corresponding subset. All X-axes use the objective lens’ scale.

**Fig 9:**
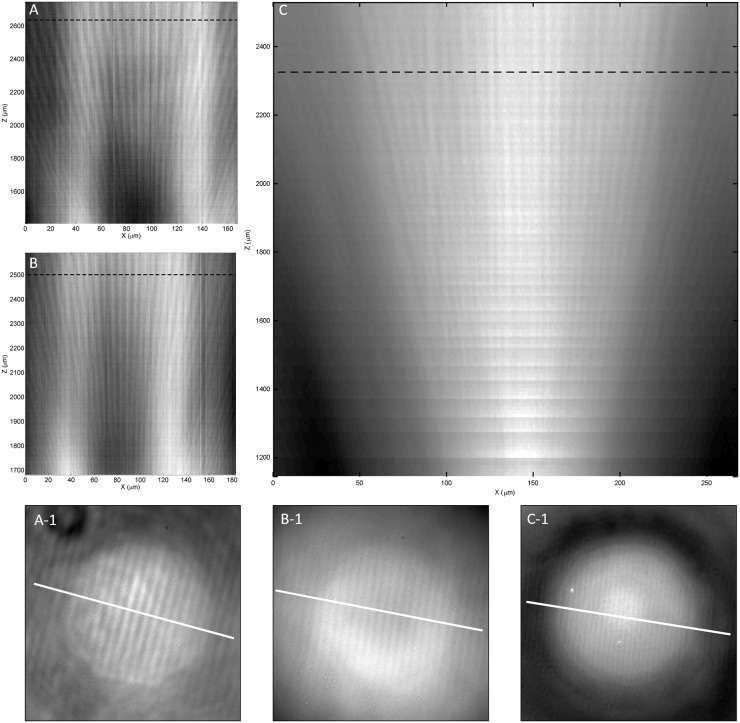
Cross-section slice of the z-stacked images captured under different conditions. (A) a 20x (NA 0.75) objective lens with a 220-μm BTG; (B) a 20x (NA 0.45) objective lens with a 220-μm BTG; and (C) a 10x (NA 0.3) objective lens with a 220-μm BTG. Subsets (A-1), (B-1), and (C-1) correspond to subfigures (A)-(C), where each shows the cutting line that results in the cross-section slice. Similarly, the dotted line in each cross-section slice shows the focus position when capturing the corresponding subset. All X-axes use the objective lens’ scale.

Images that are formed by stacking cross-sections perpendicularly to the Blu-ray disc’s lines at different focus positions, which show trends in the illumination and magnification information with the change in focus position, are shown in Fig 8. For a microsphere with a 25-μm diameter, central black spots appear throughout the range of focus at super-resolution. Conversely, a larger microsphere decreases the background gradient and thus creates a larger area with good contrast. With larger microspheres, the range of focus at super-resolution is much larger, and the maximum magnification that still contains sub-wavelength information is also larger; this allows a low-magnification objective lens to achieve sub-wavelength resolution due to sufficiently large magnification enhancement provided by a larger microsphere. In Fig 7, the last column shows the resolved Blu-ray disc features using a combination of a 220-μm-diameter microsphere and a low-magnification objective lens (10x N.A. 0.3). This result shows the unique opportunity for low-cost microscopes to be transformed into super-resolution microscopes. A conservative estimation of the useful field-of-view tells us that microspheres with a diameter as large as 220 μm, operating in real-imaging mode, can provide a field-of-view diameter as large as 9 μm, which is double what 25-μm-diameter microspheres can provide. The cross-section slices in Fig 9 show results produced with an objective lens with different numerical apertures; both a 20x objective lens with N.A. 0.75 and with N.A. 0.45 can resolve Blu-ray disc lines clearly.

However, they are not without differences. While a higher aperture values yield a starker contrast than lower aperture values, the non-uniformity of light produced is also greater; high-aperture-value objective lenses also have the disadvantage of being more sensitive to any optical defects within the light path, resulting in distortions of important features, as shown in Fig 9(A-1). In Fig 9(C), fan-like stripes are still visible and identifiable, further providing evidence that the narrow gap of 100 nm is resolvable even when using an objective lens with an N.A. as low as 0.3 and a magnification factor as low as 10x.

**Fig 10:**
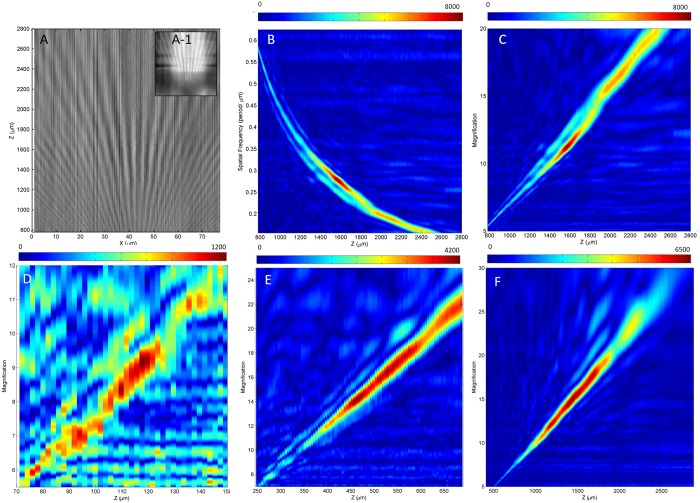
Results obtained from the cross-section of z-stacked images. (A) first difference of the cross-section of z-stacked images, where inset (A-1) shows the original cross-section using a 220μm diameter BTG; (B) Zoom FFT Analysis of (A), where the magnitude of the frequency is represented by its color; (C) magnification analysis of (B); (D) magnification analysis on 25μm diameter BTG; (E) magnification analysis on 65μm diameter BTG; (F) magnification analysis obtained on 175μm diameter BTG. The strength of the magnification is represented by the color. The results are all obtained using a 50x (NA 0.45) objective lens.

To further strengthen the relationship between additional magnification and focus position, the additional magnification factor is quantified using a robust analysis of the slices we obtained in Fig 8. As there are known periodic dimensions in a Blu-ray disc, Blu-ray disc is therefore perfect for frequency analysis. The first difference (an operation of subtracting the right neighouring pixel with each pixel) of the slice image is used for subsequent frequency analysis because this difference technique can filter any non-uniformity in low-frequency light, as shown in Fig 10(A), originating from Fig 8(D). A Zoom Fast Fourier transform (Zoom FFT) [23], which is also called a chirp Z-transform (CZT), is used in the frequency analysis process, which is known to enhance the resolution of certain parts of the frequency spectrum [23]. A 1D CZT was performed on the entire row of gray value data at each focus position. A restacking of these 1D frequency spectra is shown in Fig 10(B), for which the spatial frequency is different from that shown above, which is defined in terms of how many periods of the Blu-ray disc grooves there are within 1 micron, as observed using the microscope’s objective lens. The magnitude of each frequency at each focus position is displayed using colors. A distinct, yellowish, thin, curved area indicates an inversely proportional relationship between the spatial frequency and focus position. Because the pitch of Blu-ray disc tracks is known to be the industry standard of 320 nm [24], a color graph that plots magnification versus focus position can be obtained from Fig 10(B), resulting in Fig 10(C). The same steps can be repeated for the slice images obtained in Fig 8(A)–8(C) resulting in Fig 10(D)–10(F). Focus positions with the red areas of the color graph indicate a more concentrated distribution and also reveal the overall sharpness of that focus position. This analysis also helps us to identify the best focus position for the best resolution.

**Fig 11:**
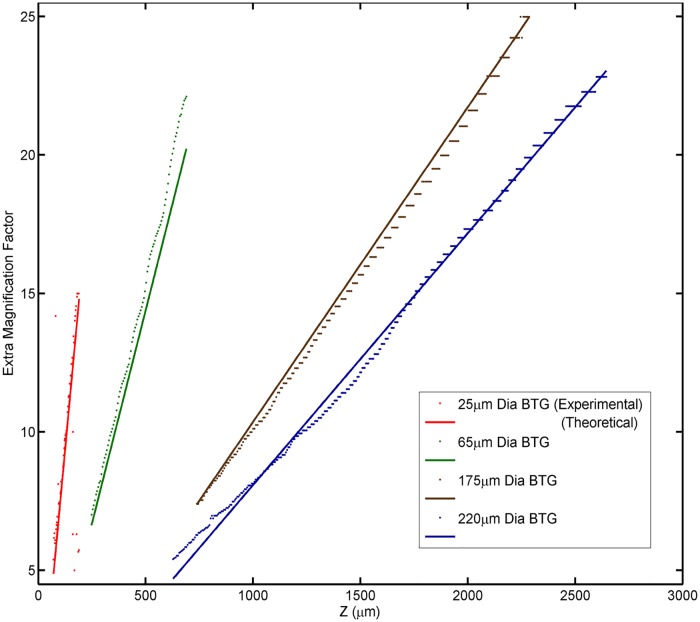
Plot of the theoretical and experimental magnification using different sizes of microspheres against the z-position.

Other than the above analysis, which is based on the experimental results, there is a theoretical way to predict the magnification using the focus position. From the FDTD simulation, a line passing through the dipole and the center point of the microsphere is an axis where the formation of the image lies, as shown in each subplot of Fig 5. It is shown that all streamlines form a symmetry with this line, proving that it is the axis of image formation. Therefore, the magnification can be easily related to the focus position using this principle and be derived to obtain the following equation:

$$\text{M}=\frac{1}{\text{R}+\text{d}}\text{z}-1\text{ z}>0$$ (1)

where M is the magnification factor contributed by the microsphere; R is the radius of the microsphere; z is the focus position, where z = 0 is the sample’s surface of interest; and d is the distance between the lowest point of the microsphere and the sample’s surface of interest. Because the microsphere is attached to the sample’s surface in all experiments in this study, it can be assumed that d = 0, and the magnification slope depends entirely on the size of the microsphere. To see if this theoretical prediction agrees with the results of the experimental analysis, the maximum-magnitude point of each row of the color graph on magnification is extracted to form a scatter plot. This kind of scatter plot is obtained for different sizes of microspheres and is combined with the theoretical plot for comparison in Fig 11. All the scatter plots were acquired using the same objective (50x 0.45). The results show that both the theoretical and experimental method agree but do show reasonable differences. The marginal difference between the two methods can be attributed to factors that include a marginally imperfect sphere, defects on the Blu-ray disc or microspheres, measurement inaccuracies of the sphere diameter and occasional inconsistencies in the dimensions in the Blu-ray disc track pitches. With such minor differences, the theoretical method can be considered to reasonably estimate the real scale of an object being observed if the focus position is known with reference to the position that focuses on the sample surface. A further evidence on the applicability of Eq (1) is shown when comparing the values of feature’s measurement by conventional objectives and the estimation from Eq (1). The brighter lines in the image captured without the microsphere in Fig 4(B) were measured to be spaced at 1.15 μm. Similar brighter lines were also observed in the image when using a 200-μm-diameter microsphere. Matching the features in both images, we arrived at a corresponding additional magnification of 6.40x when using a microsphere. This result is similar to the magnification of 6.44x, which was calculated using Eq (1) with the focus position controlled at 703 μm away from the sample’s surface.

Fig 11 shows that larger microspheres have a wider range of focus positions that can reach the sub-diffraction-limit resolution and have a smaller slope with the change in focus position. This result indicates that larger microspheres are more robust to changes or measurement errors in the focus position; this allows for more reliable estimation of the feature dimensions using large microspheres.

Despite the aforementioned advantages of large microspheres, these advantages do not imply that larger microspheres always produce better resolutions; smaller microspheres can theoretically produce better resolutions. This is indicated by our simulation results mentioned earlier. Moreover, from the evanescent waves analysis reported by Ben-Aryeh [25], surfaces that have larger angles with the sample’s surface can convert evanescent waves at higher spatial frequencies into propagating waves. However, for a spherical shape, surfaces that have larger angles with the sample are farther away from the sample’s surface; thus, due to the exponential decay of evanescent waves, the transmitted evanescent waves will have a much weaker intensity. Therefore, when the radius of the microsphere decreases, large angle surfaces are closer to the sample’s surface, and the final signal at higher spatial frequencies are stronger. Thus, while larger microspheres have a wider range of focus, less strict requirements on the objective lenses, z-stage precision and a larger field-of-view, they also have a lower resolution. As a result, the choice of microsphere size is a balance between resolution limit and various flexibilities.

## Conclusions

The discovery of real super-resolution images using high-refractive-index microspheres has been detailed above, providing a new operating mode for microsphere-assisted microscopy. While being comparable to virtual imaging modes in terms of resolution, this new mode also lowers the limit on the working distance of an objective lens and provides flexibility for additions that may have been previously hindered by this working distance. For example, a high-precision scanning arm that can be used for scanning can have a millimeter clearance even with a high-aperture-value objective lens. We also concluded, using both simulation and experimental results, that the refractive index of the microsphere used in such an application is critical to whether the image formed is virtual or real. A relationship between the focus position and the additional magnification was established both experimentally and theoretically. This theoretical relationship was proven to be similar to experimental observations, allowing for direct calculations of feature dimensions without prior calibrations for images obtained through microspheres. It was also discovered that, for the real imaging mode, larger size microspheres actually provide a larger useful field-of-view and require less accurate z-axis information to acquire dimensions. Finally, we have demonstrated that images obtained using the real imaging mode with large microspheres can still resolve sub-diffraction-limit features for an additional magnification of 10x and up. This can allow for an inexpensive, low-magnification objective lens to be used for large-field-of-view, super-resolution imaging. Together with the advantage of the simple synthetic process for producing microspheres compared to solid-immersion lens or other super-lenses, the proposed method could reduce the difficulties associated with obtaining super-resolution images.
